# Can Winged Aphid Abundance Be a Predictor of Cucurbit Aphid-Borne Yellows Virus Epidemics in Melon Crop?

**DOI:** 10.3390/v12090911

**Published:** 2020-08-20

**Authors:** Alexandra Schoeny, Loup Rimbaud, Patrick Gognalons, Grégory Girardot, Pauline Millot, Karine Nozeran, Catherine Wipf-Scheibel, Hervé Lecoq

**Affiliations:** INRAE, Pathologie Végétale, F-84140 Montfavet, France; loup.rimbaud@inrae.fr (L.R.); patrick.gognalons@inrae.fr (P.G.); gregory.girardot@inrae.fr (G.G.); pauline.millot@inrae.fr (P.M.); karine.nozeran@inrae.fr (K.N.); catherine.rys@inrae.fr (C.W.-S.); rvbalecoq@gmail.com (H.L.)

**Keywords:** *Aphis gossypii*, *Cucumis melo*, cucurbit viruses, disease progress curve, insect trapping, logistic model, Spearman correlation, temporal dynamics

## Abstract

Aphid-borne viruses are frequent yield-limiting pathogens in open field vegetable crops. In the absence of curative methods, virus control relies exclusively on measures limiting virus introduction and spread. The efficiency of control measures may greatly benefit from an accurate knowledge of epidemic drivers, in particular those linked with aphid vectors. Field experiments were conducted in southeastern France between 2010 and 2019 to investigate the relationship between the epidemics of cucurbit aphid-borne yellows virus (CABYV) and aphid vector abundance. Winged aphids visiting melon crops were sampled daily to assess the abundance of CABYV vectors (*Aphis gossypii*, *Macrosiphum euphorbiae* and *Myzus persicae*) and CABYV was monitored weekly by DAS-ELISA. Epidemic temporal progress curves were successfully described by logistic models. A systematic search for correlations was undertaken between virus variables including parameters µ (inflection point of the logistic curve) and γ (maximum incidence) and aphid variables computed by aggregating abundances on periods relative either to the planting date, or to the epidemic peak. The abundance of *A. gossypii* during the first two weeks after planting was found to be a good predictor of CABYV dynamics, suggesting that an early control of this aphid species could mitigate the onset and progress of CABYV epidemics in melon crops.

## 1. Introduction

In France, melon (*Cucumis melo* var. *cantalupensis*) is cultivated in three main production areas: South-East, South-West and Centre-West. South-East produces around 40% of the national production (224,720 t, 11,720 ha in 2019, www.agreste.agriculture.gouv.fr). Open field melon crops are frequently infected by viruses among which is cucurbit aphid-borne yellows virus (CABYV, Polerovirus, Luteoviridae). Since its first report in the 1980s [1], CABYV has been detected in an ever-increasing number of countries [2] and recent surveys indicate that it is becoming prevalent in many cucurbit growing areas [3,4]. It induces typical symptoms of yellowing of the older leaves and impacts yield via flower abortion and reduced number of fruits per plant. It is transmitted in a persistent, circulative, non-propagative manner by a few aphid species (*Aphis gossypii*, *Macrosiphum euphorbiae* and *Myzus persicae*) [5]. In melon crops, *A. gossypii* seems to be largely involved in CABYV epidemics [6,7] but it is still not clear if monitoring *A. gossypii* abundance could be used as a predictor of CABYV epidemics.

In the absence of curative methods, virus control relies exclusively on measures limiting virus introduction and spread (prophylaxis, genetic resistance, vector control, sanitation) [8]. Concerning CABYV, although resistance genes have been identified in at least two melon accessions [9,10], to our knowledge they have not yet been integrated in commercial cultivars. A recent study showed that the *Vat* gene conferring resistance to the melon aphid *A. gossypii* and the viruses it carries [11] had a significant impact on CABYV epidemics with the mean incidence reduction exceeding 50% in some trials [7]. Still, this effect is far from meeting farmer’s expectations. Therefore, complementary measures should also be employed to limit virus introduction and spread in the field. Whatever the control method under consideration (chemical, genetic, biological), its efficiency is likely to be enhanced with an improved knowledge of epidemic drivers, in particular those linked with aphid vectors. Indeed, a good understanding of the relationship between aphid vector abundance and virus epidemic dynamics will certainly help improve (i) our ability to predict future epidemics and (ii) the timeline of control measures intended to prevent the arrival and intra-field movement of aphid vectors as well as the onset and progress of viral diseases.

In this study, we investigated the relationships between aphid population dynamics and CABYV epidemics in melon crops assessed in field experiments conducted in southeastern France between 2010 and 2019. For this, we monitored both aphid populations and viral dynamics, resulting in two distinct datasets. We then looked for potential relationships between numerous variables computed from these two datasets with the overall aim of modelling CABYV epidemics using aphid abundances.

## 2. Materials and Methods

### 2.1. Field Experiments

Eleven field experiments were conducted between 2010 and 2019 in Avignon, southeastern France (Table 1): nine at the St Paul experimental station (43°54′53″ N, 4°52′59″ E) and two at the St Maurice experimental station (43°56′49″ N, 4°51′52″ E). The two sites are approximately 4 km apart. Although run in the framework of different projects, all trials involved a Charentais-type melon plot (Charentais T line, susceptible to the melon aphid *A. gossypii*) surrounded by bare soil maintained through mechanical weeding. Seedlings were prepared in an insect-proof greenhouse three weeks before planting. Depending on the trial, plants at the 1–3 leaf stage were planted in late April or late May on dark brown plastic mulch with drip irrigation. Early plantings were protected from wind and cold damage with Agryl P17 fleece (Fiberweb France, Biesheim) for 11–15 days. This row cover also protects from virus contaminations by preventing viruliferous aphids to reach the plants [8]. CABYV is not seed-borne and all plantlets grown in an insect-proof greenhouse or under Agryl P17 may be considered as healthy at the planting or fleece removal stages. The experimental plot comprised 120 to 240 plants (0.5–0.8 m plant spacing) organized in 6 to 16 rows (1.5–2 m row spacing) depending on the trial (Table 1). No insecticides were applied during the trials.

### 2.2. Plant Sampling and Virus Monitoring

In order to monitor virus dynamics, melon plants were sampled weekly, 8 times during the cropping season. Sample collections generally started one week after planting/fleece removal and investigated 13 to 60% of the crop depending on the trial (Table 1). Sampling plans were designed regardless of the presence of virus symptoms. For the sake of simplification, the nth day after planting or fleece removal will be coded as “Dn” later in the article. Expanding leaves were sampled at each sampling date. It was assumed that information obtained from an expanding leaf accurately reflects the status of the entire plant. CABYV was diagnosed via double antibody sandwich enzyme-linked immunosorbent assay (DAS-ELISA) with a specific polyclonal antiserum [1]. Virus detection was considered positive when the absorbance at 405 nm was greater than 3 times that of the mean of healthy controls.

### 2.3. Insect Sampling and Aphid Monitoring

The temporal dynamics of winged aphids visiting the melon crops were established from planting or fleece removal (D0), and lasted until the end of the virus sampling period. Winged insects were sampled at the crop height with a non-biased suction trap [12]. Catches were collected daily, rinsed and stored in 70% ethanol until sorting (aphids vs. other insects) and taxonomic identification of aphids under a stereomicroscope. Aphid abundance datasets used in this study are described in detail in [12]. The analyses focused on the three CABYV vectors reported in the literature: *A. gossypii*, *M. persicae* and *M. euphorbiae*, respectively associated with their Rothamsted Insect Survey (RIS) codes: RIS-181, RIS-322 and RIS-410.

### 2.4. Computation of Variables Related to Virus Epidemics

For each trial and sampling date, CABYV incidence was calculated as the ratio of the number of infected plants divided by the number of sampled plants. Datasets were standardized in order to facilitate data mining: when missing, incidences at D7, D14, D21, D28, D35, D42, D49 and D56 were estimated by linear interpolation from surrounding sampling dates.

CABYV epidemics were summarized by four “virus” variables. The first one is the area under the disease progress curve (AUDPC) from D0 to D56 calculated according to the formula (1): where y_i_ represents CABYV incidence, expressed as a percentage, at date D_i_.

(1) $${\mathrm{AUDPC}}_{56}=\overset{i=55}{\underset{i=0}{\sum }}\frac{\left[y_{i}+y_{i+1}\right]}{2}\times \left(D_{i+1}-D_{i}\right)$$

AUDPC_56_ was divided by the total virus monitoring duration (56 days) to calculate the mean incidence over the epidemic. On the basis of their mean incidence, epidemics were categorized as mild (0–20%), intermediate (21–40%), severe (41–60%) or extreme (61–100%) [7].

The three following virus variables are parameters of a logistic equation fitted to incidence data using nonlinear least squares (2):

(2) $$y_{t,k}=\frac{\gamma_{k}}{1+e^{-4.\alpha_{k}.\left(t-\mu_{k}\right)}}$$

where *y_t,k_* is the incidence, expressed as a percentage, at time *t* (t∈⟦1;56⟧) and for trial *k* (k∈⟦1;11⟧); *µ_k_* is the abscissa of the inflection point for trial *k*, i.e., the date of the epidemic peak. Low values of *µ* indicate precocious epidemics while high values are associated with late epidemics; *γ_k_* is the plateau, i.e., the carrying capacity, for trial *k*. High values of *γ* indicate global epidemics (high incidence at the end of the season) whereas low values mean limited epidemics; *α_k_* is related to the slope at the inflection point for trial *k*, it reflects the speed of epidemic around the peak. Roughly, high values of *α* mean fast epidemics and low values mean slow epidemics; *µ* and *α* are positive parameters; and *γ* is bounded 0 and 1.

To assess the relative influence of µ, γ and α on the average fitted virus incidence (i.e., $\bar{y}=\frac{1}{55}\overset{55}{\underset{t=1}{\sum }}y_{t}$ for a given combination of µ, γ and α), we ran a sensitivity analysis. For this, 50,000 different combinations of the three parameters were randomly drawn within their respective variation ranges (delimited by the extreme values found in the 11 trials, see Results) via a Latin hypersquare sampling method, and sensitivity indices were estimated using Sobol–Saltelli’s method [13]. The first-order index of a parameter indicates its main influence on the model output, whereas the total index also includes its interactions with other parameters. Given the negligible influence of parameter α (see Results), the following analyses focused only on AUDPC_56_, µ and γ.

### 2.5. Computation of Variables Related to Aphid Abundance

The dataset of daily aphid abundance was used to compute, for each of the three main vector species of CABYV (*A. gossypii*, *M. persicae* and *M. euphorbiae*) as well as for the total number of aphids, a wide range of aggregated “aphid” variables tested for their relationship with the virus variables. Firstly, daily abundance was aggregated on periods relative to the planting date, by calculating the sum from time t_1_ ($t_{1}\in ⟦1;55⟧$) to time t_2_ ($t_{2}\in ⟦t_{1};55⟧$), resulting in 1540 different variables for each aphid species. Secondly, daily abundance was aggregated on periods relative to the date of epidemic peak (estimated with parameter µ of the logistic curve), by calculating the sum from time t_1_ = µ − Δt_1_ ($\Delta t_{1}\in ⟦1;\mu ⟧$) to time t_2_ = µ − Δt_1_ + Δt_2_ ($\Delta t_{2}\in ⟦1;55-\text{µ}+\Delta t1⟧$). Depending on the value of µ, this resulted in a maximum of 3025 additional variables.

### 2.6. Relationship between Aphid and Virus Variables

For each of the three virus variables (AUDPC_56_, µ and γ), a relationship with one or several aphid variables was established in three steps. In a first step, we used the Spearman test with a maximal type-1 error of 1% to identify aphid variables that were significantly correlated to the virus variable under consideration. In a second step, for each remaining aphid variable, we modelled the relationship between the virus variable (dependent variable) and the aphid variable (explanatory variable). For AUDPC_56_, we used the following linear regression (3):

(3) $$z_{k}=A_{0}+A_{1}.x_{k}$$

and for µ and γ, given the shape of data, we used an exponential model (estimated using nonlinear least squares) (4):

(4) $$z_{k}=B_{0}+B_{1}\left(1-e^{-B_{2}.x_{k}}\right)$$

with:

*z_k_*—the value of the virus variable (i.e., AUDPC_56_, µ or γ) for trial *k* (k∈⟦1;11⟧);

*x_k_*—the value of the aphid variable for trial *k*;

*A*_0_ and *A*_1_—the parameters of the linear model for AUPDC_56_;

*B*_0_, *B*_1_ and *B*_2_—the parameters of the exponential model for µ and γ, such as *z_k_*(0) = *B*_0_ and *z_k_*(∞) = *B*_0_+*B*_1_.

The mean square error (MSE) was used to evaluate the goodness-of-fit of every model and thus to rank aphid variables according to their potential to explain the virus variable. Finally, in a third step, the model associated with the lowest mean square error was considered as the best candidate to relate aphid variables and the virus variable under consideration. In addition, these best candidates were used to predict values of µ and γ that were themselves used in the logistic equation to rebuild viral epidemic dynamics in each trial.

### 2.7. Data & Software

Analyses were performed using the R software version 3.5.2 [14]. The sensitivity analysis used the package “sensitivity” version 1.17.1 [15]. Aphid raw data are hosted in a public repository: Data INRAE (Dataverse). Direct URL to data is: https://doi.org/10.15454/NKRWEO.

## 3. Results

### 3.1. Virus Epidemics

CABYV was consistently detected in every trial, with epidemic types (based on mean incidence) varying from mild to extreme (Table 2).

**Figure 1:**
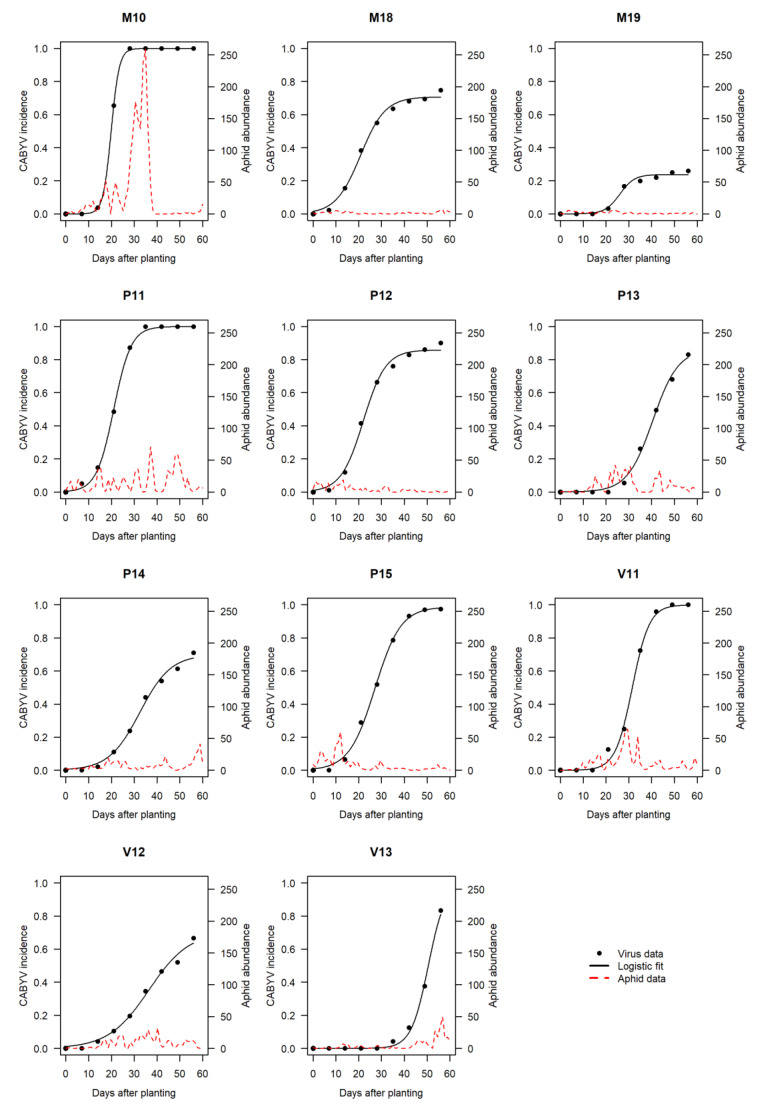
CABYV disease progress and aphid abundance assessed in melon crops in eleven field trials conducted in Avignon between 2010 and 2019. Black dots represent observed cumulative incidences (proportion of infected plants expressed as a ratio). Black solid lines are fitted curves (logistic model). Red dashed lines represent daily abundances of the pool of CABYV aphid vectors (*Aphis gossypii*, *Myzus persicae*, *Macrosiphum euphorbiae*).

CABYV disease progress curves were successfully described by the logistic model (Figure 1). Parameter µ (inflection point of the curve indicating the date at which 50% of the maximum incidence is reached) varied between 20 days (M10) and 51 days (V13) (Table 2). Parameter γ indicating the maximum incidence varied between 0.24 (M19) and 1 (M10, P11, V11, V13). Parameter α reflecting the increase rate of disease incidence per day varied between 0.028 (V12) and 0.141 (M10). Taken individually, high values of γ and α, or low values of µ do not necessarily imply severe or extreme epidemics (e.g., γ = 1 in V13 but the epidemic is mild because α is low and µ is high). There was no correlation among these parameters.

**Figure 2:**
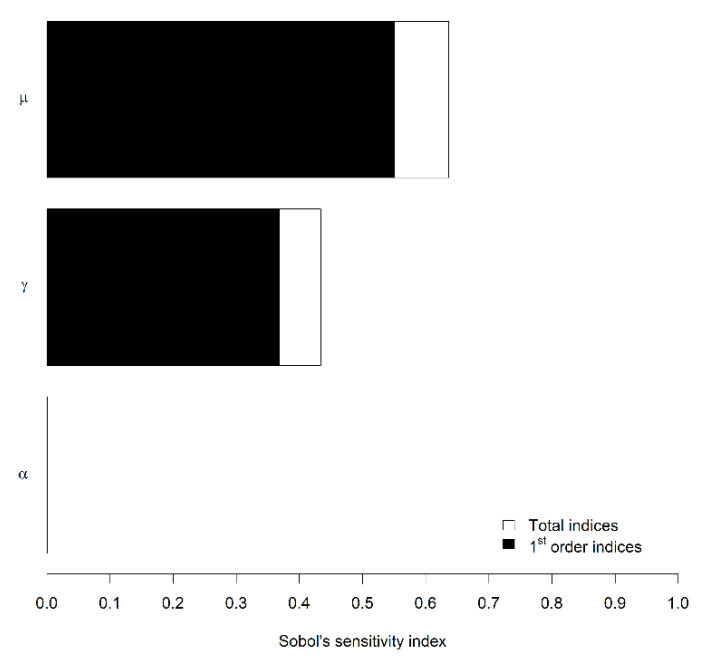
First-order and total Sobol’s sensitivity indices of the three parameters of the logistic equation on the average virus incidence. µ is the abscissa of the inflection point (i.e., the date of the epidemic peak); γ is the plateau (i.e., the carrying capacity); α is related to the slope at the inflection point (i.e., the speed of epidemic around the peak). The first-order index indicates the main influence of a parameter, whereas the total index includes its interactions with other parameters.

The average virus incidence was mostly influenced by parameters µ and γ, as indicated by their first-order Sobol’s sensitivity indices of 0.55 and 0.36, respectively (meaning that 55% and 36% of the variability in average virus incidence can be attributed to the variability in the value of µ and γ, respectively) (Figure 2). The influence of α was negligible, with a total index (which measures the influence of a parameter including its interactions with other parameters) of 0.0013.

### 3.2. Vector Abundances

The three reported CABYV vectors *A. gossypii* (RIS-181), *M. persicae* (RIS-322) and *M. euphorbiae* (RIS-410) represented generally 10% to 30% of the total aphid abundance and could exceptionally reach 52% in M10 (Table 2). *A. gossypii* and *M. persicae* were trapped in every trial. *M. euphorbiae* was present in 4 of the 11 trials and its specific abundance did not exceed three individuals per sampling campaign. With specific abundance representing up to 95% of the total vector abundance, *A. gossypii* was the most abundant vector species in all trials except V11. In V11, *M. persicae* preponderated in catches (60% of the total abundance). As for virus epidemics, patterns of aphid vector dynamics were extremely variable depending on the trials (Figure 1). In some cases, vector activity was more intense at the beginning of the crop (P15 for instance), at mid-crop (M10) or later (V13). *A. gossypii* and *M. persicae* showed dissimilar temporal patterns suggesting a dissimilar host reservoir location and/or dispersal timing (Figure S1).

### 3.3. Correlations between Virus and Aphid Variables

The large variability in both virus epidemics and aphid abundance dynamics constituted a perfect framework to study the virus–aphid link through a systematic search for correlations between three virus variables (AUDPC_56_, µ and γ; parameter α was not included because its influence on virus incidence was negligible) and more than 9000 aphid variables. These aphid variables were computed by aggregating abundances on periods relative either to the planting date, or to the date of the epidemic peak (i.e., µ, the abscissa of the inflection point of the logistic curve). Depending on the virus variable under consideration, the Spearman test yielded a diverse number of significant correlations with one or several aphid variables. For AUDPC_56_, 413 significant correlations were obtained with abundances of *A. gossypii* or the total aphid population aggregated on periods relative to the planting date (Table S1), and one correlation was obtained with *A. gossypii* abundance aggregated on a period relative to the date of epidemic peak (four consecutive days starting from 11 days before the epidemic peak) (Table S2). Parameter µ was correlated to aphid variables involving either *A. gossypii* or the total aphid population aggregated on periods of 1 to 10 consecutive days within the two first weeks of cropping (Table S1). For parameter γ, 10 significant correlations were obtained with abundances of *A. gossypii* or *M. persicae* aggregated on periods of 1 to 9 consecutive days within the three first weeks of cropping (Table S1, Figure S2A). Ten supplementary significant correlations were found with *A. gossypii* abundances aggregated on periods of 1 to 12 consecutive days before or around the inflection point (Table S2, Figure S2B).

### 3.4. Selection of the Best Aphid Variables Based on Their Potential to Explain Virus Variables

**Figure 3:**
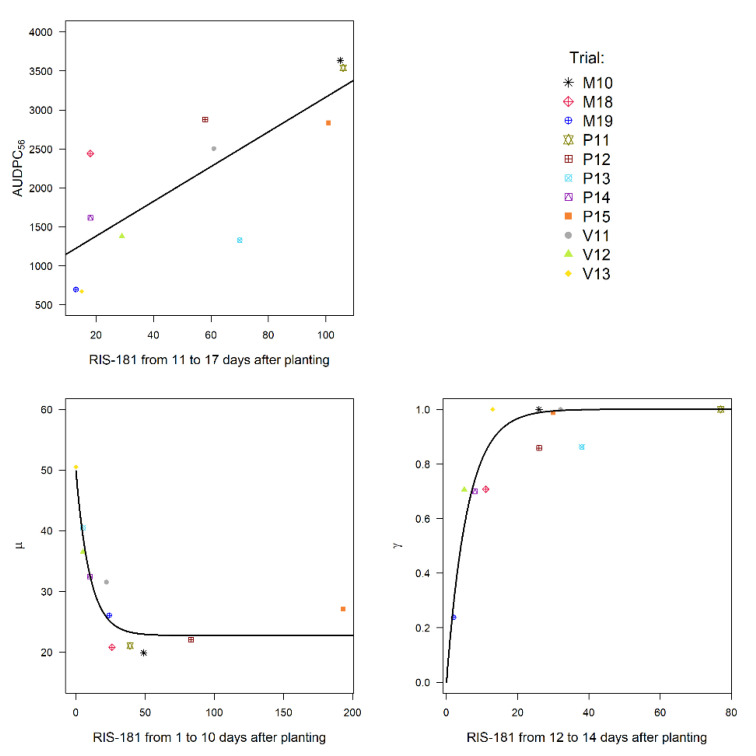
Best models obtained between the virus variables (dependent) and the aphid variables (explanatory). A linear model ($z_{k}=A_{0}+A_{1}.x_{k}$) was used for the area under the disease progress curve (AUDPC) and an exponential model ($z_{k}=B_{0}+B_{1}\left(1-e^{-B_{2}.x_{k}}\right)$) was used for two parameters of the logistic equation (µ, γ).

We used the significant correlations previously identified to build linear models to explain AUDPC_56_ and exponential models to relate µ and γ with aphid variables used as single explanatory variables. Among these models, we selected those associated with the lowest mean square error (MSE). The best linear model to explain the variability of AUDPC_56_ was obtained with the abundance of *A. gossypii* aggregated between D11 and D17 (Figure 3). The variability of parameter µ was best explained by a negative exponential model involving the abundance of *A. gossypii* aggregated between D1 and D10. With regard to parameter γ, the best exponential model involved the abundance of *A. gossypii* aggregated between D12 and D14.

### 3.5. Prediction of CABYV Epidemics

**Figure 4:**
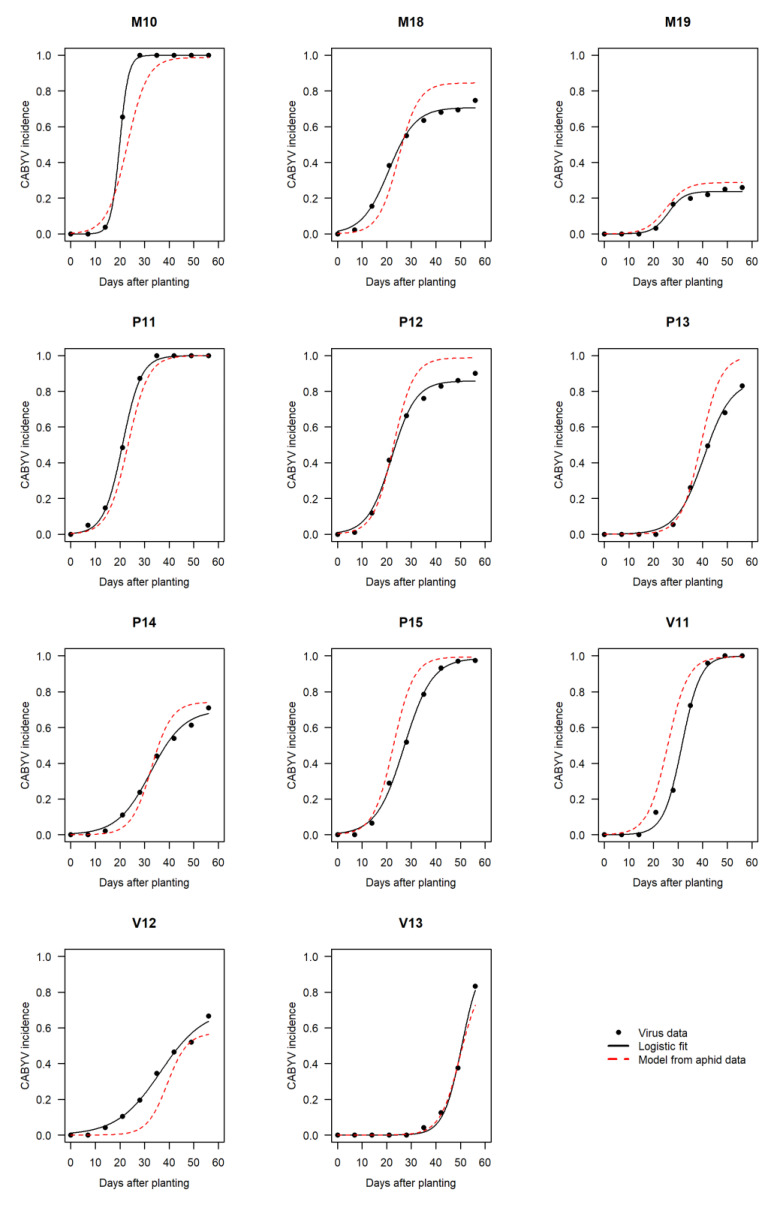
Observed and modelled CABYV epidemic dynamics in melon crops for eleven field trials conducted in Avignon between 2010 and 2019. Black dots represent observed cumulative incidences (proportion of infected plants expressed as a ratio). Black solid lines are fitted curves (logistic model). Red dashed lines represent rebuilt dynamics from the best predictive aphid variables.

The best aphid variables selected at the previous step were used to predict values of parameters µ and γ that, in turn, were used in the logistic equation to rebuild CABYV epidemics in each trial, with α fixed at its mean value 0.061 (Figure 4). The overall shapes of predicted dynamics were in agreement with observed ones and epidemic typology (mild to extreme) was generally maintained. In 6 of the 11 cases, predictions slightly overestimated the actual CABYV incidence, due to either an overestimation of parameter γ (M18, P12, P13, P14) or an underestimation of parameter µ (P15, V11). In 3 of the 11 cases, predicted and observed CABYV dynamics coincided (M19, P11, V13). In 2 of the 11 cases, predictions slightly underestimated the actual CABYV incidence, due to an overestimation of parameter µ (M10) or underestimation of parameter γ (V12).

## 4. Discussion

CABYV epidemics were observed in all eleven field experiments conducted between 2010 and 2019 in Avignon, confirming that among the viruses frequently infecting melon crops, namely cucumber mosaic virus (CMV, Cucumovirus, Bromoviridae), watermelon mosaic virus (WMV, Potyvirus, Potyviridae) and zucchini yellow mosaic virus (ZYMV, Potyvirus, Potyviridae), it has become one of the most prevalent. This situation is consistent with recent observations made in the French Mediterranean basin [3] and other countries [4]. When present, other cucurbit viruses do not seem to interfere with CABYV. For example, Schoeny et al. [7] observed a complete decoupling between the progress curves of CABYV, CMV and WMV during the cropping season, suggesting that biotic and/or abiotic factors involved in the epidemiology of these viruses are different. In the present study, CABYV progress over time (expressed as days after planting or fleece removal) was successfully described by the logistic model. This model commonly used to describe the temporal dynamics of plant viruses [16,17,18,19] has three parameters (µ, γ, α) with a biological sense (epidemic precocity, carrying capacity, epidemic speed) that were considered as dependent variables in statistical analyses and data mining. Parameters were uncorrelated. In particular, there was no correlation between µ and α, suggesting that early epidemics do not necessarily rise faster than late epidemics contrary to what has been observed in some pathosystems such as virus yellows disease in sugar beet where an increasing host resistance with plant age to feeding aphids is documented [20].

The sensitivity analysis run on the logistic model using randomly drawn combinations of these three parameters revealed that parameters µ (inflection point of the curve) and γ (maximum incidence) had a strong influence on the variability in the average CABYV incidence, whereas parameter α reflecting the disease increase rate per day had a negligible influence on the variability in virus incidence. Parameter µ appeared predominant since its variability could explain 55% of the variability in virus incidence. In our experimental conditions, values of 20–22 days for µ induced systematically severe or extreme epidemics, regardless of the value of γ. With later inflection points, epidemics could be mild to severe depending on γ. Therefore, the earliness of a virus epidemic determines greatly its destiny and consequently its impact on yield. Indeed, the earlier a plant is infected the more yield components are penalized. For CABYV, although not clearly documented, the timing of the virus epidemic compared to the flowering period is likely to be decisive in the fruit development since CABYV is known to induce flower abortion, and consequently a reduction in the number of fruits per plant and an increase in unmarketable over caliber fruits [2].

CABYV being phloem-limited, its acquisition from an infested plant and inoculation to a healthy plant require a phloem-feeding phase by the aphid vector. Contrary to viruses transmitted in a nonpersistent manner through brief intracellular probes into epidermal and/or mesophyll cells by numerous visiting aphids searching for a suitable host, CABYV is transmitted by only a few aphid species (*A. gossypii*, *M. euphorbiae* and *M. persicae*) [5]. Our study focused on this short list of potential vectors.

Winged aphids were monitored daily at the crop height with a non-biased suction trap [12]. Unlike the Rothamsted Insect Survey suction trap monitoring aphid migration flights at a height of 12.2 m above ground [21], our trap sampled winged aphids actually visiting the crop, and possibly transmitting viruses. Among the focused aphid species, *M. euphorbiae* was almost absent from catches, whereas *A. gossypii* and *M. persicae* were present in all trials, with *A. gossypii* being predominant in 10/11 trials. Aphid and virus dynamics were monitored on concomitant periods which facilitated the search for correlations between contemporary events. Although significant correlations were found with aphid variables involving both aphid species as well as total vector abundances, the best correlations involved *A. gossypii*. This is in agreement with the fact that this aphid species is the only aphid species consistently observed feeding and developing colonies on melon crops in France (Boissot, pers. com).

Among the aphid variables highly correlated to virus variables, some appeared as significant explanatory variables. Therefore, the variability of AUDPC_56_ could be explained by the abundance of *A. gossypii* aggregated between D11 and D17 using a simple linear model. The variability of µ and γ were respectively explained by the abundance of *A. gossypii* aggregated between D1 and D10, and between D12 and D14 using exponential models. It is noteworthy that these two parameters can be predicted as early as two weeks after planting/fleece removal. Thereby, using these predicted parameter values in the logistic equation, it is possible to have an early insight into the probable CABYV dynamic.

This early prediction could permit the implementation of tactical control measures destined to control *A. gossypii* populations. Among possible control measures, the use of insecticides could be optimized by guiding the positioning of treatment in time and space according to the abundance of *A. gossypii* during the two first weeks of the melon crop. Conversely, unnecessary treatments could be avoided if the abundance of *A. gossypii* during this period is low.

A disease forecast prior to planting would be of even greater usefulness. For example, Congdon et al. [22] developed an empirical model to forecast pea seed-borne mosaic virus (PSbMV) incidence in field pea crops using pre-growing season rainfall to calculate an index of aphid abundance which is used in combination with the virus infection level in the sown seed, to provide forecasts before sowing to allow sufficient time to implement control recommendations. Similarly, Steinger et al. [23] observed that the post-harvest incidence of potato virus Y (PVY) in seed potato in Switzerland could be accurately predicted by the cumulative abundance of *Brachycaudus helichrysi* (from first appearance in spring up to mid-June) and that this abundance was positively correlated to the mean daily winter temperature (January–February) indicating that winter conditions could be used as an early warning signal for PVY risk in the current season. Therefore, regarding our pathosystem, forecasting *A. gossypii* spring flying patterns as a function of winter climatic conditions could be worth investigating in order to deploy strategic control measures before planting. For example, whenever available, the selection of a resistant cultivar is an efficient and environmentally friendly way of reducing a disease risk. Concerning the melon crop, *Vat* is a gene conferring resistance to both *A. gossypii* and the viruses it carries [11]. In particular, a five-year field experiment demonstrated that *Vat* had a significant impact on CABYV epidemics with mean incidence reduction exceeding 50% in some trials [7]. Cultural practices such as the use of plastic mulches acting as an aphid repellent [24], floating row covers to prevent viruliferous aphids reaching the crop until the flowering stage or weeding to remove virus reservoirs [8] could complement this genetic resistance. Indeed, CABYV infects cucurbit crops (cucumber, melon, squash and watermelon) but also weed species such as *Capsella bursa-pastoris*, *Lamium amplexicaule* and *Senecio vulgaris*, which may be efficient alternative hosts [1] and more recently, Kassem et al. [6] suggested the importance of the weed species *Ecballium elaterium* as an alternative host and potential virus reservoir. Finally, the implementation of flower strips composed of rigorously selected plant species could also contribute to regulate the populations of aphid vectors by favoring natural enemies [25].

To conclude, our results suggest that the abundance of *A. gossypii* visiting the melon crop during the first fortnight is a good predictor of the CABYV risk, information that could be integrated in a decision support system to improve the efficiency and durability of chemical control. As recently demonstrated by Schoeny et al. [7], *A. gossypii* can also be highly involved in CMV epidemics. Therefore, early flights of *A. gossypii* represent a high virus risk but also a high infestation risk by *A. gossypii* clones capable of developing colonies on melon crops [26]. Therefore, an accurate prediction of this global risk is likely to limit economically unjustified treatments and limit their negative impact on the surrounding environment.

## Figures and Tables

**Table 1:** Melon crop and sampling details for field trials conducted in Avignon between 2010 and 2019.

Trial Code	Experimental Site	Planting Date	Number of Plants	Number of Rows	Number of Plants Per Row	Row Spacing (m)	Plant Spacing (m)	Number of Plants Sampled Per Date (Sampling Effort %)
M10	St Paul	28/05/2010	160	8	20	2	0.8	26 (16%)
V11	St Paul	09/05/2011 ^a^	120	6	20	2	0.5	24 (20%)
V12	St Paul	11/05/2012 ^a^	150	6	25	2	0.5	24 (16%)
V13	St Paul	06/05/2013 ^a^	150	6	25	2	0.5	24 (16%)
P11	St Paul	24/05/2011	208	16	13	1.5	0.5	40 (19%)
P12	St Paul	31/05/2012	240	16	15	1.5	0.5	40 (17%)
P13	St Paul	24/05/2013	240	16	15	1.5	0.5	32 (13%)
P14	St Paul	27/05/2014	240	16	15	1.5	0.5	40 (17%)
P15	St Paul	28/05/2015	240	16	15	1.5	0.5	40 (17%)
M18	St Maurice	25/05/2018	160	8	20	1.5	0.5	96 (60%)
M19	St Maurice	28/05/2019	160	8	20	1.5	0.5	96 (60%)

^a^ Agryl P17 fleece removal; fleece optimizes plant growth by increasing both air and soil temperatures and reducing wind damage.

**Table 2:** CABYV epidemics and winged aphid abundances in melon crops in eleven field trials conducted in Avignon between 2010 and 2019. Epidemics are summarized by their area under the disease progress curve calculated over 56 days (AUDPC_56_), mean incidence (AUDPC_56_/56), epidemic category and parameter estimates of the logistic models (µ, γ and α) fitted to cumulative incidences. *Aphis gossypii* (RIS-181), *Myzus persicae* (RIS-322), *Macrosiphum euphorbiae* (RIS-410) and total aphid abundances were monitored with suction traps between 1 and 55 days after planting.

Trial	AUDPC_56_	Mean Incidence (%)	Epidemic Category ^a^	µ	γ	α	RIS-181	RIS-322	RIS-410	Total Aphids
M10	3635	65	Extreme	20	1.00	0.141	1693	110	0	3468
M18	2443	44	Severe	21	0.71	0.046	90	5	0	810
M19	697	12	Mild	26	0.24	0.078	76	7	3	841
P11	3540	63	Extreme	21	1.00	0.066	776	72	0	3113
P12	2878	51	Severe	22	0.86	0.051	207	24	0	4004
P13	1330	24	Intermediate	40	0.86	0.045	506	49	0	1772
P14	1617	29	Intermediate	32	0.70	0.037	277	139	0	1382
P15	2834	51	Severe	27	0.99	0.044	407	53	0	2271
V11	2502	45	Severe	32	1.00	0.067	256	379	1	2488
V12	1379	25	Intermediate	36	0.71	0.028	317	118	2	4097
V13	671	12	Mild	51	1.00	0.066	246	46	3	1468

^a^ On the basis of their mean incidence, epidemics were categorized as mild (0–20%), intermediate (21–40%), severe (41–60%) or extreme (61–100%) [7].

## Supplementary Materials

The following materials are available online at https://www.mdpi.com/1999-4915/12/9/911/s1, Figure S1: Daily abundance of CABYV aphid vectors monitored in melons crops with non-biased suction traps in eleven field trials conducted in Avignon between 2010 and 2019. Aphis gossypii (RIS-181) in black solid line; Myzus persicae (RIS-322) in red dashed lines; Macrosiphum euphorbiae (RIS-410) in blue dotted lines, Figure S2: Significant correlations between parameter γ (carrying capacity of the logistic model) and aphid variables calculated: (A) on periods relative to the planting date (i.e., from t1 to t2 days after planting); (B) on periods relative to the date of epidemic peak (i.e., from t1 = µ − Δt1 to t2 = µ − Δt1 + Δt2). Horizontal bars represent the periods over which aphid abundances are aggregated. RIS-181: Aphis gossypii; RIS-322: Myzus persicae; RIS-410: Macrosiphum euphorbiae; total: total aphid population. Correlations are ranked according to the mean square error (MSE) of the corresponding models, Table S1: Significant correlations (with a maximal type-1 error of 1%) between virus variables and aphid variables computed on periods relative to the planting date. Significant correlations involve abundances of Aphis gossypii (RIS-181), Myzus persicae (RIS-322) and total aphid population (total) aggregated between t1 and t2 (in days from planting date). The relationship between the virus variable and the aphid variable was modelled with a linear model (*z_k_* = *A*_0_ + *A*_1_ · *x_k_*) for the area under the disease progress curve (AUDPC_56_) and an exponential model ($z_{k}=B_{0}+B_{1}\left(1-e^{-B_{2}.x_{k}}\right)$) for the parameters of the logistic equation (µ and γ). For AUDPC_56_, only the 15 correlations having the lowest mean square errors are presented (total of 413), Table S2: Significant correlations (with a maximal type-1 error of 1%) between virus variables and aphid variables computed on periods relative to the date of epidemic peak (µ). Significant correlations involve abundances of *Aphis gossypii* (RIS-181) aggregated between t_1_ = µ − Δt_1_ and t_2_ = µ − Δt_1_ + Δt_2_ (in days from planting date). The relationship between the virus variable and the aphid variable was modelled with a linear model ($z_{k}=A_{0}+A_{1}.x_{k}$) for the area under the disease progress curve (AUDPC_56_) and an exponential model ($z_{k}=B_{0}+B_{1}\left(1-e^{-B_{2}.x_{k}}\right)$) for the parameter γ of the logistic equation. No aphid variable was found significantly correlated to µ.
