# Supplementary material for: Can Winged Aphid Abundance Be a Predictor of Cucurbit Aphid-Borne Yellows Virus Epidemics in Melon Crop?
Source: Viruses. 2020 Aug 20;12(9):911. doi: 10.3390/v12090911 (PMC7551915; doi:10.3390/v12090911)

Figure S1. Daily abundance of CABYV aphid vectors monitored in melons crops with non-biased suction traps in eleven field trials conducted in Avignon between 2010 and 2019. *Aphis gossypii* (RIS-181) in black solid line; *Myzus persicae* (RIS-322) in red dashed lines; *Macrosiphum euphorbiae* (RIS-410) in blue dotted lines.

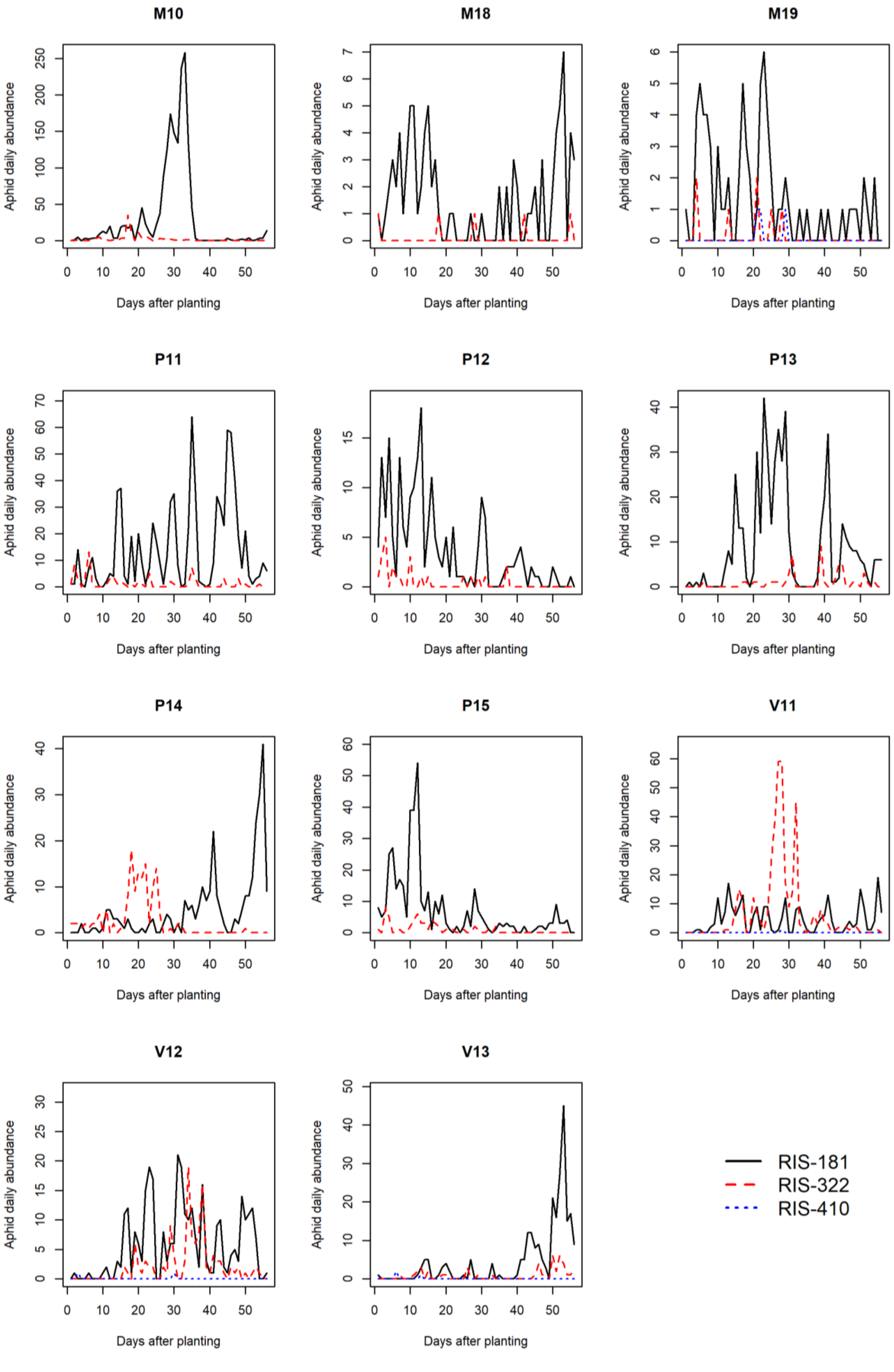

Supplement: Supplementary file 1 [file viruses-12-00911-s001.zip › Figure S1 Schoeny et al.pdf]
