# Supplementary material for: Can Winged Aphid Abundance Be a Predictor of Cucurbit Aphid-Borne Yellows Virus Epidemics in Melon Crop?
Source: Viruses. 2020 Aug 20;12(9):911. doi: 10.3390/v12090911 (PMC7551915; doi:10.3390/v12090911)

Figure S2. Significant correlations between parameter γ (carrying capacity of the logistic model) and aphid variables calculated: A) on periods relative to the planting date (i.e. from t_1 to t_2 days after planting); B) on periods relative to the date of epidemic peak (i.e. from $t_1 = \mu - \Delta t_1$ to $t_2 = \mu - \Delta t_1 + \Delta t_2$). Horizontal bars represent the periods over which aphid abundances are aggregated. RIS-181: *Aphis gossypii*; RIS-322: *Myzus persicae*; RIS-410: *Macrosiphum euphorbiae*; total: total aphid population. Correlations are ranked according to the mean square error (MSE) of the corresponding models.

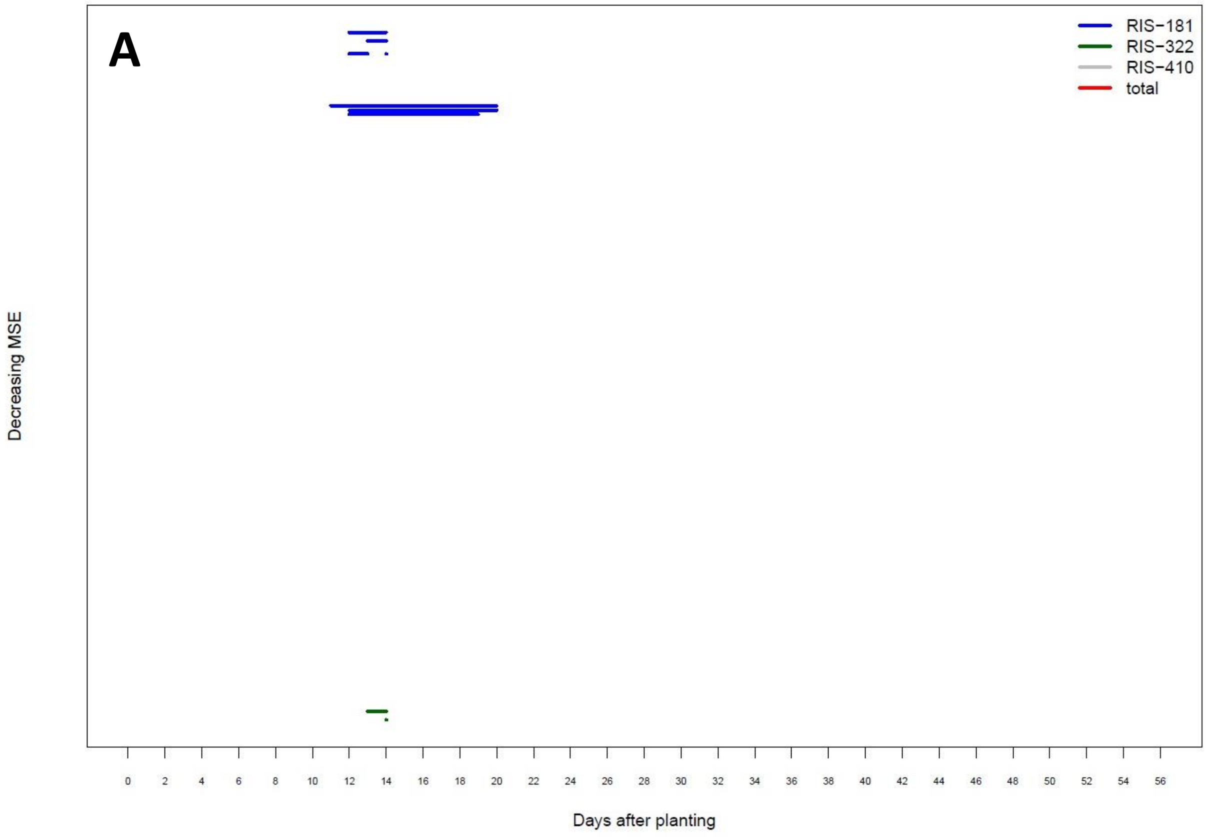

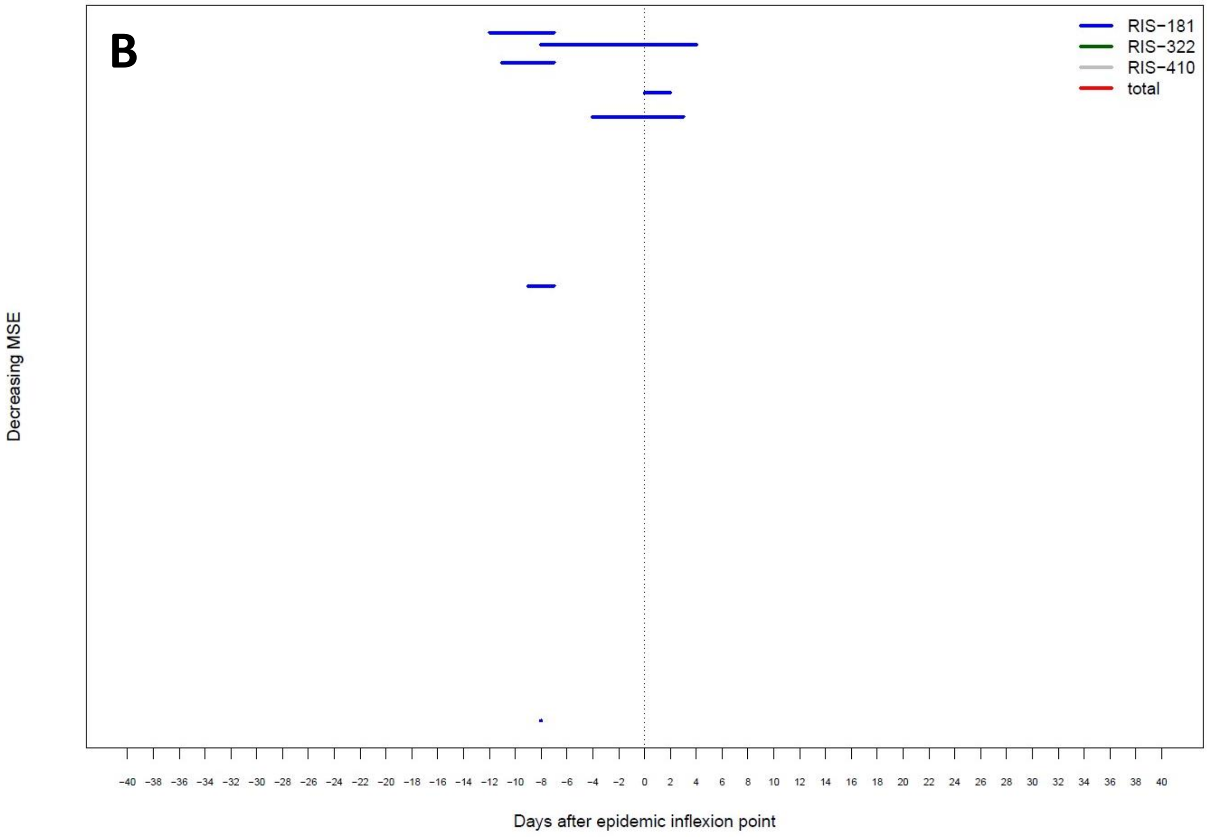

Supplement: Supplementary file 1 [file viruses-12-00911-s001.zip › Figure S2 Schoeny et al.pdf]
