# Supplementary material for: Can Winged Aphid Abundance Be a Predictor of Cucurbit Aphid-Borne Yellows Virus Epidemics in Melon Crop?
Source: Viruses. 2020 Aug 20;12(9):911. doi: 10.3390/v12090911 (PMC7551915; doi:10.3390/v12090911)
Supplement: Supplementary file 1 [file viruses-12-00911-s001.zip › Table S1 Schoeny et al.pdf]

Table S1. Significant correlations (with a maximal type-1 error of 1%) between virus variables and aphid variables computed on periods relative to the planting date. Significant correlations involve abundances of *Aphis gossypii* (RIS-181), *Myzus persicae* (RIS-322) and total aphid population (total) aggregated between t_1 and t_2 (in days from planting date). The relationship between the virus variable and the aphid variable was modelled with a linear model ($z_k = A_0 + A_1.x_k$) for the area under the disease progress curve ($AUDPC_{56}$) and an exponential model ($z_k = B_0 + B_1(1 - e^{-B_2.x_k})$) for the parameters of the logistic equation (μ and γ). For $AUDPC_{56}$, only the 15 correlations having the lowest mean square errors are presented (total of 413).

Virus variable (dependent)	Aphid variable (explanatory)	t_1	t_2	Spearman correlation coefficient	A_0	A_1	B_0	B_1	B_2	Mean square error
$AUDPC_{56}$	RIS-181	11	17	0.779	935.3	22.3	na	na	na	374278
$AUDPC_{56}$	total	4	34	0.873	794.4	0.9	na	na	na	381970
$AUDPC_{56}$	total	6	34	0.873	795.8	0.9	na	na	na	382619
$AUDPC_{56}$	total	7	34	0.855	788.6	1.0	na	na	na	383680
$AUDPC_{56}$	total	5	34	0.873	808.0	0.9	na	na	na	384915
$AUDPC_{56}$	total	8	34	0.873	790.8	1.0	na	na	na	386144
$AUDPC_{56}$	RIS-181	10	19	0.815	985.2	16.5	na	na	na	387200
$AUDPC_{56}$	total	9	34	0.873	803.6	1.0	na	na	na	391689
$AUDPC_{56}$	total	3	34	0.855	806.8	0.9	na	na	na	393733
$AUDPC_{56}$	RIS-181	10	17	0.761	997.7	18.7	na	na	na	393753
$AUDPC_{56}$	total	1	34	0.855	854.3	0.8	na	na	na	402753
$AUDPC_{56}$	total	2	34	0.836	845.6	0.8	na	na	na	404972
$AUDPC_{56}$	total	4	35	0.845	800.9	0.9	na	na	na	405497
$AUDPC_{56}$	total	10	34	0.845	831.1	1.0	na	na	na	405838
$AUDPC_{56}$	total	4	33	0.855	834.8	0.9	na	na	na	406846
μ	RIS-181	1	10	-0.797	na	na	49.9	-27.2	0.100	8.5
μ	RIS-181	2	10	-0.797	na	na	49.0	-26.1	0.106	9.3
μ	RIS-181	5	10	-0.761	na	na	50.1	-27.0	0.147	9.5
μ	RIS-181	4	10	-0.788	na	na	49.8	-26.5	0.142	9.5
μ	RIS-181	3	10	-0.799	na	na	49.6	-26.5	0.119	10.2
μ	total	2	2	-0.802	na	na	49.9	-26.1	0.262	12.0
μ	RIS-181	1	9	-0.756	na	na	48.9	-25.6	0.139	13.0
μ	RIS-181	2	8	-0.738	na	na	46.7	-24.1	0.165	13.4
μ	RIS-181	2	9	-0.764	na	na	48.1	-24.6	0.157	13.6
μ	RIS-181	4	9	-0.735	na	na	49.8	-25.9	0.268	13.7
μ	total	1	9	-0.747	na	na	54.7	-30.7	0.016	13.9
μ	RIS-181	4	11	-0.738	na	na	51.4	-28.4	0.101	14.5
μ	RIS-181	6	8	-0.789	na	na	42.4	-20.1	0.276	15.7
μ	total	6	6	-0.763	na	na	41.2	-21.4	0.036	29.7
γ	RIS-181	12	14	0.753	na	na	0	1	0.170	0.008
γ	RIS-181	13	14	0.811	na	na	0	1	0.231	0.010
γ	RIS-181	12	13	0.737	na	na	0	1	0.266	0.013
γ	RIS-181	14	14	0.735	na	na	0	1	0.530	0.013
γ	RIS-181	11	19	0.746	na	na	0	1	0.053	0.025
γ	RIS-181	11	20	0.746	na	na	0	1	0.050	0.025
γ	RIS-181	12	20	0.758	na	na	0	1	0.056	0.026
γ	RIS-181	12	19	0.735	na	na	0	1	0.060	0.027
γ	RIS-322	13	14	0.766	na	na	0	1	1.557	0.165
γ	RIS-322	14	14	0.810	na	na	0	1	1.916	0.167
