# Supplementary material for: Can Winged Aphid Abundance Be a Predictor of Cucurbit Aphid-Borne Yellows Virus Epidemics in Melon Crop?
Source: Viruses. 2020 Aug 20;12(9):911. doi: 10.3390/v12090911 (PMC7551915; doi:10.3390/v12090911)
Supplement: Supplementary file 1 [file viruses-12-00911-s001.zip › Table S2 Schoeny et al.pdf]

Table S2. Significant correlations (with a maximal type-1 error of 1%) between virus variables and aphid variables computed on periods relative to the date of epidemic peak (μ). Significant correlations involve abundances of *Aphis gossypii* (RIS-181) aggregated between $t_1 = \mu - \Delta t_1$ and $t_2 = \mu - \Delta t_1 + \Delta t_2$ (in days from planting date). The relationship between the virus variable and the aphid variable was modelled with a linear model ($z_k = A_0 + A_1.x_k$) for the area under the disease progress curve ($AUDPC_{56}$) and an exponential model ($z_k = B_0 + B_1(1 - e^{-B_2.x_k})$) for the parameter γ of the logistic equation. No aphid variable was found significantly correlated to μ.

Virus variable (dependent)	Aphid variable (explanatory)	Δt_1	Δt_2	Spearman correlation coefficient	A_0	A_1	B_0	B_1	B_2	Mean square error
$AUDPC_{56}$	RIS-181	11	3	0.764	725.9	54.1	na	na	na	369776
γ	RIS-181	12	5	0.774	na	na	0	1	0.067	0.027
γ	RIS-181	8	11	0.735	na	na	0	1	0.038	0.029
γ	RIS-181	8	12	0.735	na	na	0	1	0.037	0.029
γ	RIS-181	11	3	0.772	na	na	0	1	0.091	0.032
γ	RIS-181	11	4	0.777	na	na	0	1	0.079	0.032
γ	RIS-181	0	2	0.750	na	na	0	1	0.162	0.037
γ	RIS-181	0	3	0.781	na	na	0	1	0.130	0.041
γ	RIS-181	4	6	0.735	na	na	0	1	0.063	0.041
γ	RIS-181	9	2	0.758	na	na	0	1	0.198	0.069
γ	RIS-181	8	0	0.753	na	na	0	1	0.501	0.141
